# Oral vinorelbine plus cisplatin with concomitant radiotherapy as induction therapy for stage III non‐small cell lung cancer: Results of a single‐arm prospective cohort study

**DOI:** 10.1111/1759-7714.13125

**Published:** 2019-07-05

**Authors:** Ping‐Chih Hsu, John Wen‐Chang Chang, Chun‐Chieh Wang, Chen‐Te Wu, Yu‐Ching Lin, Chih‐Liang Wang, Tin‐Yu Lin, Shih‐Hong Li, Yi‐Chen Wu, Scott Chih‐Hsi Kuo, Cheng‐Ta Yang, Chien‐Ying Liu, Chih‐Hung Chen

**Affiliations:** ^1^ Division of Thoracic Medicine, Department of Internal Medicine Chang Gung Memorial Hospital at Linkou, Chang Gung University College of Medicine Taoyuan City Taiwan; ^2^ Department of Oncology Chang Gung Memorial Hospital Linkou branch Taoyuan City Taiwan; ^3^ Division of Radiation Oncology Chang Gung Memorial Hospital at Linkou, Chang Gung University College of Medicine Taoyuan City Taiwan; ^4^ Department of Radiology Chang Gung Memorial Hospital at Linkou, Chang Gung University College of Medicine Taoyuan City Taiwan; ^5^ Division of Thoracic Oncology, Department of Respiratory and Critical Care Medicine Chang Gung Memorial Hospital, Chiayi Branch Chiayi county Taiwan; ^6^ Department of Respiratory Care Chang Gung University of Science and Technology, Chiayi Campus Chiayi county Taiwan; ^7^ Division of Thoracic and Cardiovascular Surgery, Department of Surgery Chang Gung Memorial Hospital at Linkou, Chang Gung University College of Medicine Taoyuan City Taiwan; ^8^ Department of Respiratory Therapy, College of Medicine Chang Gung University Taoyuan City Taiwan

**Keywords:** Cisplatin, concurrent chemoradiotherapy, oral vinorelbine, stage III non‐small cell lung cancer, survival

## Abstract

**Background:**

Concurrent chemoradiotherapy (CCRT) is an optimal recommended treatment for stage III non‐small cell lung cancer (NSCLC). Herein, we aimed to investigate the efficacy and safety of oral vinorelbine plus cisplatin with concomitant radiotherapy for stage III NSCLC.

**Methods:**

This prospective, open‐label, single‐arm, observational cohort study was performed between January 2010 and September 2016. Patients were treated with two cycles of chemotherapy with 60 mg/m^2^ intravenous cisplatin on day 1 and 50 mg/m^2^ oral vinorelbine on days 1, 8, and 15; radiotherapy was administered concurrently from day 1 when chemotherapy was initiated. A total dose of 66–70 Gy radiotherapy was delivered in daily fractions of 2 Gy for 6.5–7 consecutive weeks. The tumor response was assessed after completing concomitant treatment.

**Results:**

A total of 58 patients were enrolled and analyzed; 31 patients had stage IIIA NSCLC and 27 had stage IIIB NSCLC. After induction CCRT, 31 patients achieved an objective response (complete response in one and partial response in 30; the response rate was 53.4%). The median progression‐free survival was 6.73 months (95% confidence interval [CI], 5.42–7.91), duration of response was 12.30 months (95% CI, 5.59–19.01), and overall survival was 24.83 months (95% CI, 19.26–30.21). No treatment‐related mortality was observed, and neutropenia was the most common grade 3 and 4 treatment‐related toxicity (11 patients; 18.9%).

**Conclusions:**

CCRT with the weekly regimen of oral vinorelbine plus triweekly cisplatin was effective and safe for stage III NSCLC.

## Introduction

Non‐small‐cell lung cancer (NSCLC) accounts for approximately 85% of lung cancer cases, and is the main cause of cancer‐related death worldwide. Approximately one‐third of NSCLC patients present with locally advanced stage III disease at initial diagnosis.^1^, ^2^ Stage III NSCLC patients present with heterogeneous medical conditions, which range from locally and extensively unresectable tumors to apparently resectable tumors with occult microscopic mediastinal nodal metastases and bulky or multistation mediastinal nodal disease.^3^ The combination of chemotherapy (CT) and radiotherapy (RT) is optimal and the recommended therapeutic strategy for locally advanced stage III NSCLC.^3^, ^4^, ^5^ Furthermore, in a phase II clinical trial, researchers tested cisplatin (CDDP)‐based combinations with a third‐generation agent—including gemcitabine (GEM), paclitaxel (PCT), and vinorelbine (NVB)—as induction CT followed by concurrent chemoradiotherapy (CCRT) with the same agents for the induction therapy of unresectable stage III NSCLC.^6^ The three agents had similar efficacy when combined with CDDP, and these combinations had response rates ranging from 67% to 74% and one‐year survival rates from 62% to 68%.^6^ However, the three agents had different safety profiles, and the CDDP‐NVB combination had less toxicity than the other two combinations, especially with regard to grade 3–4 esophagitis (NVB, 25%; PCT, 39%; and GEM, 52%).^6^

Oral vinorelbine (NVBo) is a relatively new formulation, and NVBo and intravenous NVB have similar treatment efficacy and safety profiles for advanced NSCLC.^7^, ^8^, ^9^ NVBo and CDDP have been used as induction therapy followed by NVBo and CDDP with concomitant RT for stage III NSCLC; NVBo combined with CDDP was effective and well‐tolerated (with a treatment completion rate of 87%) in stage III NSCLC patients.^10^, ^11^ CCRT may be a more effective strategy than sequential chemoradiotherapy for locally advanced NSCLC.^12^ However, the efficacy and safety of NVBo in neoadjuvant CCRT for locally advanced stage III NSCLC are under investigation in eastern Asian patients. Therefore, this study aimed to evaluate the efficacy and safety profile of CDDP plus NVBo in combination with RT for locally advanced stage III NSCLC.

## Methods

### Patients

This prospective, open‐label, single‐arm, observational cohort study was approved by the institutional review board (No. 99‐1900A3). Patients in the study were enrolled from two centers: the Chang Gung Memorial Hospital in Linkou and the Chang Gung Memorial Hospital in Chiayi. All patients provided signed written informed consent before enrollment. The inclusion criteria were as follows: histologically and/or cytologically confirmed locally advanced stage III NSCLC; age between 20 and 80 years; surgically unresectable tumor as reviewed by radiologists, surgeons, and oncologists on a tumor board; at least one measurable lesion according to the RECIST criteria; Eastern Cooperative Oncology Group performance status (ECOG‐PS) score ≤2; weight loss ≤10% within the past three months prior to screening; life expectancy ≥12 weeks per the judgment of investigators; hemoglobin ≥10 g/dL, neutrophils ≥1.5 × 10^9^/L, or platelets ≥100 × 10^9^/L; total bilirubin ≤1.5 × upper limit normal (ULN), transaminases <2.5 × ULN, or alkaline phosphatases <5 × ULN; and creatinine <1.4 mg/dL or creatinine clearance rate >60 ml/min. Patients were excluded if they had previously undergone surgery for NSCLC or had received RT, systemic CT, targeted therapy, or other anticancer drugs. The intent‐to‐treat (ITT) population was defined as all enrolled patients who received at least one dose of CDDP plus NVBo in combination with RT, and the ITT population was used for the evaluation of efficacy and safety.

The sample size was estimated assuming that the response rate of the treatment group and reference group would be 60% and 50%, respectively. A difference of 10% in response rates was considered to have no clinical significance. Thus, under a two‐sided significance level of 0.05 and power of 80%, the estimated sample size was 84 patients.

### Treatment plan

**Figure 1:**
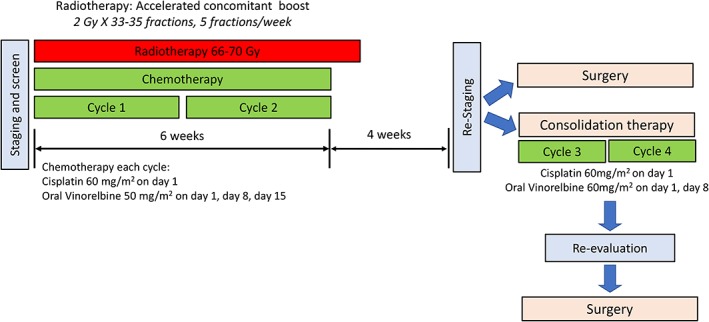
Treatment scheme of the study.

Bioequivalence studies have shown that 60 and 80 mg/m^2^ NVBo is equivalent to 25 and 30 mg/m^2^ of the intravenous formulation, respectively.^7^, ^9^ The dose of CDDP and NVBo was adjusted according to two previous studies and the ethnicity of the patients in our study, all of whom were eastern Asian.^10^, ^13^ NVBo was administered at a dose of 50 mg/m^2^ on days 1, 8, and 15, and CDDP was delivered intravenously at a dose of 60 mg/m^2^ on day 1. RT was administered concurrently with the first two cycles of CT. A total dose of 66–70 Gy was delivered in daily fractions of 2 Gy for 6.5–7 consecutive weeks. The clinical RT target area consisted of the primary volume of the gross tumor and node, ipsilateral hilum region, and elective mediastinum for which the lower edge was 3.0 cm below the carina. The planning radiation target area was a margin plus the clinical RT target area to ensure that the prescribed dose precisely covered the lesion. The tumor response was assessed after completing the second cycle of concomitant treatment. Patients who achieved an objective response (complete response [CR]/partial response [PR]) after induction therapy and were willing to undergo surgery were referred to the lung cancer tumor board for presurgery evaluation. Patients underwent resection if the lung cancer tumor board deemed surgery feasible after reviewing the clinical and imaging data. Patients who achieved an objective response (CR/PR) or stable disease (SD) but who could not undergo surgery received two additional cycles of consolidation therapy consisting of 60 mg/m^2^ NVBo on days 1 and 8 and 60 mg/m^2^ CDDP on day 1. Some patients who achieved an objective response after consolidation therapy and could undergo surgery after being re‐evaluated by the lung cancer tumor board also underwent resection. All the patients were covered by the national health insurance (NHI) program of Taiwan for the treatments including CT, RT, and surgery. Treatment was discontinued if the disease progressed or if adverse effects were observed. The treatment plan is summarized in Figure 1.

### Efficacy and safety evaluations

The parameters evaluated included response rate, time to disease progression, overall survival (OS), duration of response (DOR), and toxicity profile. The imaging tests used for treatment efficacy evaluation were computed tomography, positron emission tomography, and chest radiography. Computed tomography was performed at baseline, weeks 3–7 of the second CCRT cycle, and the end‐of‐study visit. Positron emission tomography was performed at baseline and during the preoperation evaluation for determining which tumors were potentially resectable after downstaging. Chest radiography was performed at baseline and at the investigating physician's discretion during follow‐up. Other imaging studies such as bone scintigraphy were also performed at the investigating physician's discretion if clinically indicated. The treatment response was assessed according to version 1.1 of the Response Evaluation Criteria in Solid Tumors (RECIST). The objective response rate (CR + PR) was the primary endpoint of this study. The secondary endpoints of this study were progression‐free survival (PFS), DOR, and OS. The time to disease progression was measured from the date of initial combination therapy to the date of the first disease progression or death. Patients who had not shown disease progression or died at the time of study completion and those who were lost to follow‐up were considered censored on the date of the last tumor assessment. The DOR was measured from the first date on which CR or PR was observed to the first date when recurrent or progressive disease (PD) or death was documented. Patients with no documented progression after CR or PR were censored at the last date on which they were known to have had the CR or PR, respectively. The OS was defined as the time from the date of combination therapy to the date of death, regardless of the cause of death. Patients who were alive at the time of study completion were censored at the date of the last follow‐up assessment. Survival and disease progression data were analyzed until December 2017. If death or progression had not occurred, the survival time and progression time were considered censored.

Safety evaluations included a physical examination as well as evaluating the vital signs, ECOG‐PS, complete blood cell counts, serum biochemistry, and clinical safety. Toxicities and adverse events were graded according to the National Cancer Institute Common Terminology Criteria for Adverse Events (CTCAE) version 3.0.^14^

### Statistical analysis

The demographic and baseline characteristics were presented as the number for qualitative variables or median and range for quantitative variables as appropriate. Survival analysis was performed by using the Kaplan‐Meier method, and the data were summarized as the number of cases, number of censored cases, median time point estimate, and the 95% CI for the median value. Treatment‐related toxicity was coded by using a coding system, and the results were summarized in a frequency table by using categorization, causality, severity, and number of patients with at least one adverse event. All statistical analyses were performed by using GraphPad Prism (Version 5.0; GraphPad Software, San Diego, CA, USA).

## Results

A total of 58 patients with locally advanced stage III NSCLC were enrolled between January 2010 and September 2016. Patient characteristics are shown in Table 1.

**Table 1:** Baseline characteristics of the patients

	No.	(%)
Total included	58	
Sex		
Male	51	88
Female	7	12
AGE median (range)	61 (33–79)	
ECOG PS		
0	4	7
1	54	93
Smoking status		
Non‐smoker	10	17
Former + current smoker	48	83
Histology		
Adenocarcinoma	14	24
Squamous cell carcinoma	36	62
Other NSCLC	8	14
Stage		
IIIA	31	53
IIIB	27	47
Median time from diagnosis to study entry, median days (range)	10 (5–34)	

During induction CCRT, treatment was discontinued in three patients because of PD. Among the three patients who underwent incomplete CCRT, one patient received one cycle of CT and 3500 cGy RT, while the other two received two cycles of CT and 6000 cGy RT. The remaining 55 patients underwent the complete CCRT course, with CT treatment delay observed in 34 patients. In the 34 patients who experienced CT treatment delay, treatment delay occurred on day 8 during cycle 1 in one patient, on day 15 during cycle 1 in nine patients, on day 1 during cycle 2 in five patients, on day 8 during cycle 2 in five patients, and on day 15 during cycle 2 in 16 patients. Hematological toxicity was the main reason (24 events, 70%) for CT treatment delay, followed by suspected infections (seven events, 21%), patient decision (two events, 6%), and deterioration in renal function (one event, 3%).

A total of 40 patients received consolidation CT after induction CCRT, and 17 treatment delay events were noted during the consolidation CT course. Among the 17 treatment delay events, treatment delay occurred on day 8 during cycle 3 in three patients, on day 1 during cycle 4 in five patients, and on day 8 during cycle 4 in the remaining nine patients. Hematological toxicity was the main reason for treatment delay in eight patients (47%), and infection was the reason for treatment delay in six patients (35%). The reasons for treatment delay in the remaining three patients, included the patient's decision in one case, grade 3 abnormal aspartate aminotransferase (AST) levels in another, and pulmonary embolism in one patient.

### Response and efficacy

**Figure 2:**
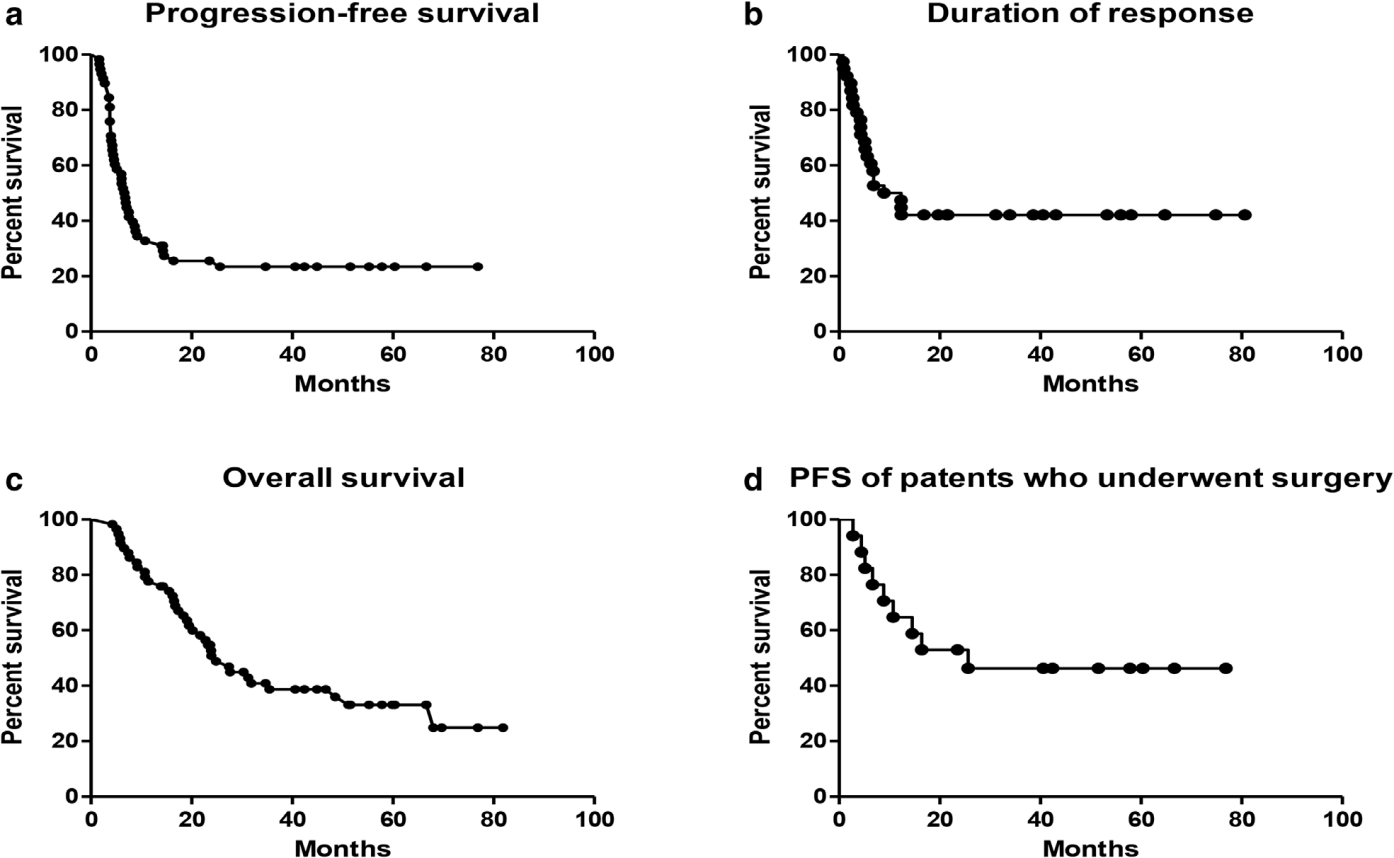
Median progression‐free survival (PFS), duration of response (DOR), and overall survival (OS) of the intent‐to‐treatment (ITT) population. (**a**) The median PFS was 6.73 months (95% confidence interval [CI], 5.42–7.91). Median PFS = 6.73 months (**b**) The median DOR was 12.30 months (95% CI, 5.59–19.01). Median DOR = 12.3 months. (**c**) The median OS was 24.83 months (95% CI, 19.26–30.21). Median OS = 24.83 months. (**d**) The median PFS of patients who underwent surgery was 25.67 months (95% CI, 25.41–57.39). Median PFS = 25.67 months.

**Figure 3:**
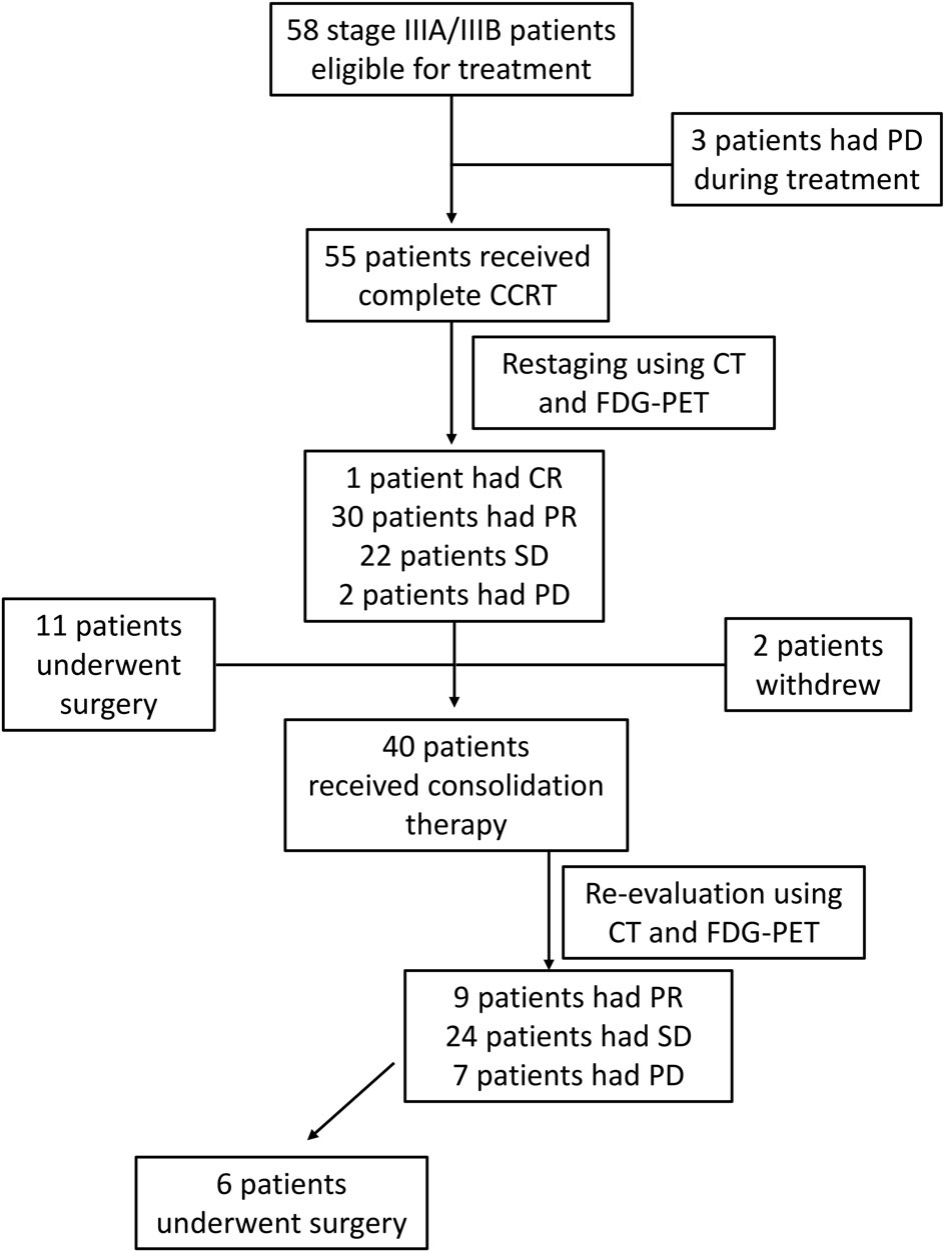
CONSORT diagram of this study.

The response evaluation was analyzed in the ITT population (58 patients). One patient achieved CR and 30 patients achieved PR, and the overall objective response rate was 53.4%. A total of 22 patients (38%) had SD, and five (8.6%) had PD (Table 2). The median PFS of the ITT population was 6.73 months (95% CI, 5.42–7.91; Fig 2a). For treatment responders, the median DOR was 12.30 months (95% CI, 5.59–19.01; Fig 2b). The median OS was 24.83 months (95% CI, 19.26–30.21; Table 2; Fig 2c). The 29 patients with PD within seven months were further analyzed (Table S1). Metastasis was the most common cause of disease progression (58.5%), followed by clinical progression (24.1%) and local progression (20.7%). Among the 29 patients, 24 (82.8%) received at least one‐line of systemic therapies including platinum‐base doublet CT, single agent CT, and epidermal growth factor receptor (EGFR)‐tyrosine kinase inhibitor (TKI) after disease progression (Table S2). The characteristics and clinical information of the 17 patients who underwent surgery are shown in Table 3. The study consort diagram is shown in Figure 3.

**Table 2:** Treatment response and outcomes of concurrent chemoradiotherapy (CCRT) and surgery

Total		58
CR		1
PR		30
SD		22
PD		5
Overall response rate (%)		31/58 (53.4%)
Median PFS (months)		6.73 (95% CI [5.42–7.91])
Median DOR (months)		12.30 (95% CI [5.59–19.01])
Median survival (months)		24.83 (95% CI [19.26–30.21])
Patients received surgery after downstaging (%)		17 (29.3%)
Types of surgery		
Pneumonectomy		1
Lobectomy		14
Segmentectomy		1
Wedge resection		1
Resection score		
R0		17
Nodal downstaging (to N1 or N0)		15
Pathological complete remission		7

CR, complete response; DOR, duration of response; PD, progressive disease; PFS, progression‐free survival; PR, partial response; SD, stable disease.

**Table 3:** Characteristics of the 17 patients who underwent surgery

				Tentative stage					Pathological nodal stage		
No.	Age	Sex	Histology	T	N	Stage	FEV1(L)	Thoracostomy	N	Outcome	Note
1	55	Male	Squamous	3	1	IIIA	2.79	Pneumonectomy	0	Dead	Brain metastatic recurrence
2	50	Male	Squamous	4	3	IIIB	2.08	Lobectomy	0	Alive	Pathological complete remission
3	58	Male	NSCLC	2	2	IIIA	3.29	Lobectomy	0	Alive	Pathological complete remission
4	71	Male	Adenocarcinoma	3	2	IIIA	1.5	Lobectomy	0	Dead	Intestinal metastatic recurrence
5	62	Female	Adenocarcinoma	3	2	IIIA	2.88	Lobectomy	0	Alive	
6	52	Male	Squamous	3	3	IIIB	2.84	Lobectomy	0	Alive	
7	72	Male	Squamous	3	2	IIIA	1.93	Lobectomy	0	Dead	Die of pneumoniae
8	62	Male	Squamous	3	3	IIIB	2.87	Lobectomy	1	Dead	Local recurrence
9	79	Male	Squamous	2	2	IIIA	1.81	Wedge resection	0	Alive	Pathological complete remission
10	64	Male	Squamous	4	1	IIIB	1.8	Lobectomy	0	Alive	Pathological complete remission
11	61	Male	Squamous	3	3	IIIB	3.11	Lobectomy	0	Alive	Pathological complete remission
12	50	Male	Adenocarcinoma	2	2	IIIA	2.72	Segmentectomy	0	Dead	Pathological complete remission
											Recurrence with brain metastasis
13	64	Male	Adenosquamous	2	2	IIIA	1.8	Lobectomy	0	Alive	Pathological complete remission
14	33	Female	Adenocarcinoma	4	2	IIIB	2.08	Lobectomy	2	Alive	Local recurrence
15	61	Male	Squamous	4	2	IIIB	2.66	Lobectomy	0	Alive	
16	50	Male	Squamous	4	2	IIIB	2.58	Lobectomy	0	Dead	Local recurrence
17	69	Female	Squamous	3	1	IIIA	1.41	Lobectomy	2	Dead	Brain metastatic recurrence

### Safety

Among hematological toxicities, the only grade 3 and 4 adverse event was neutropenia (11 patients; 18.9%). No patient in this study experienced treatment‐related grade 3 and 4 anemia or thrombocytopenia. Only one patient experienced febrile neutropenia during treatment (Table 4). Among non‐hematological toxicities, one patient each had grade 3 vomiting and grade 3 fatigue. Grade 3 abnormal AST elevation was observed in this study, but it recovered after treatment interruption (Table 4). A total of 20 patients had grade 1 and 2 RT‐related pneumonitis. Two patients had grade 3 radiation pneumonitis and were treated with systemic steroid and other supportive care (oxygen); this complication was manageable and did not worsen. No treatment‐related mortality occurred in this study.

**Table 4:** Treatment‐related toxicities

Toxicity	Grade 1 & 2	Grade 3 & 4
Hematological		
Neutropenia	20	11
Thrombocytopenia	27	0
Anemia	33	0
Febrile neutropenia	1	
Non‐hematological		
Nausea and/or vomiting	18	1
Diarrhea	16	0
Constipation	22	0
Esophagitis	6	0
Fatigue	35	1
Alopecia	2	0
Skin rash	4	0
Abnormal AST/ALT	17	1
Radiation pneumonitis	20	2

## Discussion

The results of our observational study suggest that NVBo plus CDDP with concomitant RT (i.e., CCRT) is effective and safe for unresectable stage III NSCLC. NVBo plus CDDP with CCRT yielded a response rate of 53.4%, and the median OS was 24.83 months. In addition, no treatment‐related death was observed and the main grade 3 and 4 treatment‐related toxicity was neutropenia (18.9%).

The management of stage III NSCLC patients is complex, and a multi‐modality treatment approach including CT in combination with RT or surgery is the optimal strategy.^4^, ^15^ CCRT may be better than sequential chemoradiotherapy in improving the survival of locally advanced NSCLC patients.^16^, ^17^ Theoretically, CT could induce the cell cycle to stay in the more radiation‐sensitive G2/M phase and enhance radiation‐induced cytotoxicity in cancer cells, thereby suggesting that CCRT is more effective for treating locally advanced NSCLC.^18^, ^19^ NVB was as effective as gemcitabine and paclitaxel when combined with CDDP, and in fact, had a more preferred safety profile.^6^, ^19^ Moreover, NVBo in combination with CDDP is effective for stage III NSCLC.^9^, ^10^, ^11^, ^12^, ^13^, ^20^, ^21^ The combination of CDDP and NVBo with optimal RT doses was safe with high rates of treatment completion (87%) and good efficacy. During the concomitant phase, 40 mg/m^2^ NVBo was administered once a day on days 1 and 8, every three weeks, and 80 mg/m^2^ CDDP was administered triweekly.^10^ In another study, 50 mg/m^2^ NVBo on days 1, 8, and 15 over four weeks in combination with 20 mg/m^2^ CDDP on days 1–4 was feasible in combination with RT.^13^ Thus, on the basis of the results of these two studies, we adjusted the CDDP dose to 60 mg/m^2^ every three weeks and 50 mg/m^2^ NVBo was administered once a day on days 1, 8, and 15 every three weeks considering the factors of efficacy and ethnicity (all patients were eastern Asian).

The overall response rate in our study was higher than that reported by Descourt *et al*. (53.4% vs. 41%) and similar to that reported by Krzakowski *et al*. (53.4% vs. 53.7%).^10^, ^11^ However, the PFS was 6.7 months in this study, which was shorter than that reported in three other studies using the same CT regimen concomitant with RT.^11^, ^20^, ^21^ The PFS was 15 months for elderly patients in the study by Locher *et al*., 9.2 months in the study by Descourt *et al*., and 14 months in the study by Lerouge *et al*. In the study by Locher *et al*., 40 patients were included which is smaller than the number of patients in our study (*n* = 58), and the elderly patients included were strictly selected via a comprehensive geriatric assessment (CGA) with highly clinical stability.^20^ In contrast, most of the patients (93%) in our study had ECOG PS scores of one, indicating that they at least had some clinical symptoms (e.g., dyspnea and cough). These clinical symptoms may partly limit the physical activity of patients at the time of entry in our study, possibly being the reason for the shorter PFS in our study compared to the PFS in the study by Locher *et al*. The patients in the studies of Descourt *et al*. and Lerouge *et al*. were pretreated with induction systemic CT, indicating that the patients would have had at least SD status after induction CT prior to receiving CCRT.^11^, ^21^ However, in our study, metastasis accounted for the most common cause of disease progression (58.5%), followed by clinical progression (24.1%) and local progression (20.7%) in the 29 patients whose disease progressed within seven months. In fact, 24 of the 29 (82.8%) patients received systemic therapies after disease progression. Thus, this may explain why despite the short PFS in our study (6.7 months), the OS of our study was not inferior to those in the other three studies (24.8 vs. 21.8 vs. 20.8 vs. 17.1 months).

To our knowledge, our study is the first to show the efficacy and safety of NVBo plus CDDP with concomitant RT for pan‐eastern Asian patients with stage III NSCLC. Previous studies^10^, ^11^, ^13^, ^20^, ^21^ were mainly carried out on European patients, while the present study was performed on eastern Asian patients. Taxanes (e.g., paclitaxel and docetaxel) are frequently used in combination with CDDP or carboplatin during CCRT for stage III NSCLC.^6^, ^22^, ^23^, ^24^ Hematological grade 3 and 4 neutropenia is a major complication during CCRT and often delays and interrupts treatment. In more severe cases, grade 3 and 4 neutropenia can lead to treatment‐related death.^24^ In most studies on platinum‐based regimens combined with taxanes, the incidence of grade ≥ 3 neutropenia was often higher than 40%.^6^, ^15^, ^22^, ^23^, ^24^ In a previous study on NVBo plus CDDP with concomitant RT, treatment‐related death occurred at induction CT with CDDP plus docetaxel.^11^ In contrast, in the current study, there was no treatment‐related death, which was similar to another study in which NVBo plus CDDP was used in CCRT and induction therapy.^6^

Resection is feasible for selected patients with stage III NSCLC who achieved PR or SD after neoadjuvant CCRT. Patients who underwent lobectomy after neoadjuvant CCRT had better survival than those who received definitive CCRT only.^15^, ^25^ In the current study, 17 patients underwent resection after induction CCRT or consolidation therapy. One patient decided to opt for pneumonectomy. The median PFS of patients who underwent surgery was 25.67 months. Considering the post‐operative pathological findings, 15 patients had nodal downstaging and seven had pathological complete remission, six of whom are currently alive and disease‐free. Thus, the results of the present study provide some perspectives regarding the treatment of patients with stage III NSCLC. First, NVBo plus CDDP with concomitant RT may be a potentially curative treatment for stage III NSCLC. Second, surgery may improve the outcomes with better local control and survival among selected patients with stage III NSCLC who received induction CCRT. All patients who underwent surgery in our study were reviewed and evaluated by multiple modality tumor boards, as selected patients with stage III NSCLC benefit from surgery after multidisciplinary evaluation and management.^26^, ^27^

The role of consolidation CT after CCRT for locally advanced NSCLC still remains unclear. Consolidation CT may improve OS of patients with locally advanced NSCLC receiving CCRT, but not the response rate and PFS.^28^, ^29^ Moreover, analysis failed to show evidence that CCRT provided any significant survival benefit for locally advanced NSCLC.^30^ However, our study commenced in January 2010, when the role of consolidation CT after CCRT was being investigated in several studies.^22^, ^31^, ^32^, ^33^ Therefore, in our study, consolidation CT was administered in most patients whose tumors were inoperable after CCRT.

In conclusion, NVBo and CDDP with concomitant RT (i.e., CCRT) is an effective treatment for stage III NSCLC, with an acceptable toxicity profile. Surgery is feasible for selected patients who received CT with NVBo and CDDP as part of CCRT.

## Disclosure

No authors report any conflict of interest.

## Author contributions

The study was conceived and designed by Chih‐Hung Chen, Chien‐Ying Liu, and Cheng‐Ta Yang. Data including patient referral, treatment planning, etc was acquired by all the authors. Ping‐Chih Hsu and Chien‐Ying Liu analysed and interpreted the data. Ping‐Chih Hsu and Cheng‐Ta Yang wrote, reviewed and revised the manuscript. The study was supervised by Chih‐Hung Chen, Chien‐Ying Liu, and Cheng‐Ta Yang.

## Supporting information


**Table S1** Characteristics of 29 patients with progression‐free survival (PFS) of <7 months.

**Table S2** Sites of progression and treatment for the 29 patients with progression‐free survival (PFS) of <7 months.
