# Supplementary material for: Oral vinorelbine plus cisplatin with concomitant radiotherapy as induction therapy for stage III non‐small cell lung cancer: Results of a single‐arm prospective cohort study
Source: Thorac Cancer. 2019 Jul 5;10(8):1683–91. doi: 10.1111/1759-7714.13125 (PMC6669803; doi:10.1111/1759-7714.13125)
Supplement: Supplementary file 1 — Table S1 Characteristics of 29 patients with progression‐free survival (PFS) of <7 months. [file TCA-10-1683-s001.docx]

**Supplementary table S1. Characteristics and clinical information of 29 patients whose PFS shorter than 7 months**

|  | Age | Sex | Histology | Stage | Cause of PD | Treatment following CCRT PD | Survival condition |
| --- | --- | --- | --- | --- | --- | --- | --- |
| No.1 | 47 | M | Adeno | IIIB | Neck LN metastasis | Platinum-base chemotherapy, | Dead |
| No.2 | 55 | M | SCC | IIIA | Brain metastasis | Platinum-base chemotherapy, Radiotherapy to brain | Dead |
| No.3 | 66 | M | SCC | IIIA | Clinical progression  (No site identified) | Platinum-base chemotherapy | Alive |
| No.4 | 60 | M | Adeno | IIIA | Lung to lung metastasis | EGFR-TKI | Dead |
| No.5 | 69 | F | Adeno | IIIB | Brain metastasis | Radiotherapy to brain | Dead |
| No.6 | 60 | M | SCC | IIIB | Pleural metastasis | Supportive care | Dead |
| No.7 | 57 | M | SCC | IIIA | Local recurrence | Platinum-base chemotherapy | Dead |
| No.8 | 57 | M | SCC | IIIB | Lung to lung metastasis | Platinum-base chemotherapy | Dead |
| No.9 | 70 | M | SCC | IIIA | Clinical progression  (No site identified) | Platinum-base chemotherapy | Dead |
| No.10 | 48 | M | NSCLC | IIIA | Clinical progression  (No site identified) | Platinum-base chemotherapy | Alive |
| No.11 | 71 | M | SCC | IIIA | Local progression | Single agent chemotherapy | Dead |
| No.12 | 71 | M | SCC | IIIB | Liver | Platinum-base chemotherapy | Dead |
| No.13 | 61 | M | Adeno | IIIB | Clinical progression  (No site identified) | EGFR-TKI | Dead |
| No.14 | 71 | M | SCC | IIIA | Clinical progression  (No site identified) | Platinum-base chemotherapy | Alive |
| No.15 | 65 | M | SCC | IIIA | Adrenal metastasis | Single agent chemotherapy | Dead |
| No.16 | 62 | F | SCC | IIIB | Local recurrence | Platinum-base chemotherapy | Dead |
| No.17 | 60 | M | NSCLC | IIIB | Bone | Platinum-base chemotherapy  Radiotherapy to bone | Dead |
| No.18 | 71 | M | Adeno | IIIA | Adrenal metastasis | Platinum-base chemotherapy | Dead |
| No.19 | 57 | M | SCC | IIIA | Clinical progression  (No site identified) | Platinum-base chemotherapy | Alive |
| No.20 | 51 | M | NSCLC | IIIB | Local recurrence+ pleural effusion | Platinum-base chemotherapy | Dead |
| No.21 | 62 | M | SCC | IIIB | Clinical progression  (No site identified) | Platinum-base chemotherapy | Dead |
| No.22 | 64 | M | SCC | IIIB | Supraclavicular LN metastasis | Platinum-base chemotherapy | Dead |
| No.23 | 63 | M | ADENO | IIIA | Brain metastasis | Radiotherapy to brain | Dead |
| No.24 | 73 | F | SCC | IIIA | Brain metastasis | Platinum-base chemotherapy, radiotherapy to brain | Alive |
| No.25 | 64 | M | NSCLC | IIIA | Brain metastasis | Platinum-base chemotherapy, radiotherapy to brain | Dead |
| No.26 | 69 | F | SCC | IIIA | Brain metastasis | Single agent chemotherapy, Radiotherapy to brain | Dead |
| No.27 | 73 | M | NSCLC | IIIB | Local progression | Supportive care | Dead |
| No.28 | 58 | M | ADENO | IIIA | Brain metastasis | Single agent chemotherapy, Radiotherapy to brain | Dead |
| No.29 | 64 | M | SCC | IIIB | Local progression | Supportive care | Dead |

*EGFR-TKI: Epidermal growth factor receptor tyrosine kinase inhibitor
