# Supplementary material for: Oral vinorelbine plus cisplatin with concomitant radiotherapy as induction therapy for stage III non‐small cell lung cancer: Results of a single‐arm prospective cohort study
Source: Thorac Cancer. 2019 Jul 5;10(8):1683–91. doi: 10.1111/1759-7714.13125 (PMC6669803; doi:10.1111/1759-7714.13125)
Supplement: Supplementary file 2 — Table S2 Sites of progression and treatment for the 29 patients with progression‐free survival (PFS) of <7 months. [file TCA-10-1683-s002.docx]

| **Sites** | **N (%)** |
| --- | --- |
| Local progression and recurrence | 6 (20.7) |
| Lung to lung metastasis | 2 (6.9) |
| Pleura | 2 (6.9) |
| Brain | 7 (24.1) |
| Liver | 1 (3.4) |
| Adrenal | 2 (6.9) |
| Bone | 1 (3.4) |
| Distant lymph nodes | 2 (6.9) |
| Clinical progression  (No site identified) | 7 (24.1) |
| **Treatment following CCRT PD** | **N (%)** |
| Systemic treatment |  |
| Platinum-base chemotherapy | 18 (62.1) |
| Single agent chemotherapy | 4 (13.8) |
| EGFR-TKI | 2 (6.9) |
| Local radiotherapy to brain or bone | 7 (24.1) |
| Supportive care | 3 (10.3) |

**Supplementary table S2 Sites of progression and following treatment after progression in the 29 patients with PFS shorter than 7 months**
